# *Francisella tularensis* - Immune Cell Activator, Suppressor, or Stealthy Evader: The Evolving View from the Petri Dish

**DOI:** 10.4172/2157-2526.1000144

**Published:** 2016-04-12

**Authors:** Kristen M. Holland, Sarah J. Rosa, Karsten R.O. Hazlett

**Affiliations:** Center for Immunology & Microbial Disease, Albany Medical College, Albany, NY 12208, USA

**Keywords:** Tularemia, Host-adaptation, Active suppression, Cytokines, Host-response

## Abstract

One of the hallmarks of pulmonary tularemia, which results from inhalation of *Francisella tularensis* - a significant bioterrorism concern, is the lack of an acute T_H_1-biased inflammatory response in the early phase of disease (days 1–3) despite significant bacterial loads. In an effort to understand this apparent hypo-responsiveness, many laboratories have utilized *in vitro* cell-based models as tools to probe the nature and consequences of host cell interactions with *F. tularensis*. The first uses of this model suggested that mammalian host cells recognize this bacterium principally through TLR2 to evoke a robust, classical T_H_1-biased cytokine response including TNF, IL-6, IL-1β, and IFN-γ. Others used this model in concert with a variety of non-genetic perturbations of the bacterial-host cell interaction and suggested that *F. tularensis* actively-suppressed the cellular response. Consistent with this notion, others engaged this model to assess isogenic mutants and, in many cases, found the mutant bacteria to be more pro-inflammatory than their WT counter-parts. Frequently, these observations were interpreted as evidence for the immunosuppressive function of the gene of interest. However, recently appreciated roles of the health of the bacterium and the impact of host factors have refined this model to suggest a “stealthy” mode of bacterial-host cell interaction (rather than one involving active suppression) consistent with the observations during early phase disease.

## Introduction

*Francisella tularensis* (Ft) is a gram-negative, facultative-intracellular bacterium that is the causative agent of tularemia, a zoonotic disease which can have mortality rates of 40–60% in untreated pneumonic infections [1]. Human virulent strains of the bacteria, such as SchuS4 (S4), are capable of causing disease via inhalation of as few as 10 CFU. These characteristics as well as being easily aerosolized have contributed to Ft’s classification as a Tier 1 select agent. Mammals infected with Ft generally do not exhibit symptoms for several days, suggesting a lack of immune stimulation by the bacteria; this incubation period is then followed by acute pneumonic symptoms. The cytokine response during Ft infection seems to parallel this observation as increased levels of cytokines such as TNF, IL-6, and IFN-γ are not readily detected in a murine pneumonic model prior to ~ day 4 [2]. Mice then begin to experience hypercytokinemia and succumb to challenge shortly thereafter [3]. The septic T_H_1 cytokine storm that immediately precedes death is believed to be induced by severe tissue damage and increased levels of host damage markers such as HMGB1 and S100A9 [4,5] and not a result of direct cell-bacterium interaction [6]. In contrast to the T_H_1 cytokines, the T_H_17 cytokines (IL-17, IL-23) along with IL-10 and TGF-β are induced at early time points indicating that the bacterium is actively recognized during the early phases of disease [7–9]. Commonly used lab strains include the attenuated live vaccine strain (LVS) and the related species *Francisella novicida* (Fn), both of which cause potentially lethal pneumonic disease in a mouse model. Ft is capable of evading and replicating within both phagocytic (macrophages [MΦ] and dendritic cells [DC]) and non-phagocytic cells (epithelial cells) in the lung [10]. Replication within these cells is known to require phagosome escape by an unknown mechanism followed by exponential replication within the cytoplasm before induction of autophagy and eventual cell death. Efforts to understand the mechanisms of Ft infection began with simplified *in vitro* systems that have now evolved to better mimic the host environment as well as utilize bacteria that are more relevant to *in vivo* infection.

## Early Experiments

One of the early goals of the field was to use *in vitro* systems to understand the results of Ft’s initial interactions with isolated immune cells. When LVS propagated in Muller Hinton Broth (MHB, the prevailing medium at the time for cultivation of Ft) were used to infect MΦs, increased mRNA levels of pro-inflammatory cytokines (TNF, IL-6, IFN-γ, IL-1β) were observed beginning at 4 hours post-infection, followed by increased protein secretion at 12–20 hours post-infection. This response was found to be TLR2-dependent [11,12] and reduced by the ability of the bacteria to escape the phagosome [13]. Other work in monocyte infection models also suggested that LVS induces an early inflammatory response that subsequently subsides [14,15]. Interestingly, the rapid responses seen *in vitro* seemed at odds with the absence of these same T_H_1 cytokines observed in animal infection models of early pulmonary tularemia. These apparently puzzling observations, suggesting that Ft activates a T_H_1 pro-inflammatory response from infected cells, were noted and confirmed by many independent groups.

## Evidence of Active Suppression

Concurrent with the above experiments, other labs searched for active suppression of the immune response by Ft. Active suppression by bacteria is generally mediated by effector proteins that are injected into the target cell via a molecular needle and specifically modulate host responses. Classically, bacterial effector proteins are either i) enzymes that directly modify specific host cell proteins through addition or removal of small molecules such as phosphate or adenylate or ii) non-enzymatic proteins that bind to endogenous enzymes to modify their activity [16]. While Ft lacks a type 3 secretion system, classically used to deliver such effectors, the bacterium does encode a functional type 6 secretion system (T6SS) [17]. In other bacteria T6SSs have been shown to target either mammalian cells or competing environmental bacteria [18,19]. Broadly speaking, Ft researchers have taken two experimental approaches to examine the potential for active suppression by Ft. The first involved assessing the ability of Ft to dampen a response to an exogenous pro-inflammatory agonist whereas the second was to genetically mutate the bacterium with the goal of knocking out immune-inhibitory functions.

### Inhibition of inflammatory stimulus

Several groups have tested Ft’s ability to suppress immune activation by an otherwise pro-inflammatory stimulus, such as *E. coli* LPS (Ec LPS) or the TLR2 agonist P3C, and have shown that Ft infection of MΦs dampens their ability to respond to subsequent or simultaneous agonist stimulation. This apparent suppression is insensitive to chloramphenicol (an inhibitor of bacterial protein synthesis) but is not apparent with killed bacteria [14–20]. Loss of the inhibitory effect upon killing of the bacteria may suggest active suppression; however, there is no increase in cytokine production in response to the killed bacteria alone which would be anticipated if live bacteria were required to dampen the cellular response [21]. Similar observations were seen with S4 infection and its ability to dampen a response elicited by Ec LPS [22]. Further, it was found that sterile S4 cultured media was also able to reduce pro-inflammatory responses to Ec LPS in DCs, and this response was preserved when Ft LPS was depleted from the cultured media [23]. However, it was later found that S4 lipids were capable of inhibiting IL-12p40 production in DCs as well as neutrophil recruitment in response to Ec LPS *in vivo* [24]. The same group subsequently found that purified capsule was able to dampen MΦ cytokine responses to P3C (a synthetic TLR2 agonist) and to an inflammatory capsule mutant of S4. Taken together, it appears that select Ft structural components have the ability to passively temper pro-inflammatory responses.

### Genetic mutation of suppressors

In an effort to identify specific Ft suppressors, several groups generated mutants of Ft in an attempt to pinpoint genes responsible for the lack of an inflammatory response in MΦs infected by Ft. LVSΔ*mviN* is missing a putative lipid II (an LPS precursor) flippase and these bacteria triggered induction of AIM2-dependent IL-1β production in MΦs [25]. LVSΔ*ripA* were also shown to stimulate inflammasomes and pro-inflammatory cytokine secretion [26]. These experiments lead researchers to believe that the proteins encoded by these Ft genes were responsible for actively suppressing the immune response. LVSΔFTL0325 is another mutant strain that was recently characterized as being attenuated in MΦs and inducing a pro-inflammatory response. FTL0325 was reported to be a surface-exposed outer membrane protein that suppressed TLR2 stimulation, interfered with NF-κB signalling, and blunted IL-1β production [27,28]. In a seminal study [29], the Monack group analysed an array of pro-inflammatory Ft mutants including *mviN*, *ripA*, *fopA*, *wbtA*, *lpxH*, and FTT0584 all of which provoked release of IL-1β in an AIM2-dependant manner from MΦs. Further analysis by scanning EM, the use of a clever bacterial-lysis reporter plasmid, and additional cytokine (TNF, IL-6) measurements revealed that all the pro-inflammatory mutants displayed aberrant morphologies and an elevated propensity to lyse in the cytoplasm of host cells. The authors i) concluded that Ft strains do not actively suppress inflammasome functions and ii) cautioned Ft researchers to carefully consider the integrity of the bacterium when interpreting experimental data. Along conceptually-similar lines, Robertson et al. [30] convincingly demonstrated that FTL0325, a lipoprotein with homology to the periplasmic peptidoglycan binding domain of OmpA, lacks surface-exposure and contributes to cell viability, morphology, and structural integrity [30]. The authors argued that loss of these functions, not a loss of FTL0325-mediated active suppression, underlies the pro-inflammatory nature of the ΔFTL0325 strain.

## Healthy Bacteria and Inclusion of Host Factors

While several Ft groups were using genetic or agonist-based approaches to probe the inflammatory/suppressive nature of Ft in cell-based *in vitro* assays, others sought to modify the assay to yield results consistent with those observed *in vivo*. Loegering et al. [21] performed *in vitro* experiments in which they observed a pro-inflammatory response in MΦs infected with LVS grown in MHB, similar to many previous reports. However, they also harvested LVS from infected MΦs and used these bacteria to infect naïve MΦs and observed very little pro-inflammatory cytokine secretion. These findings were extended to an *in vivo* model in which mice infected with MΦ-grown LVS showed increased morbidity compared to those infected with MHB-grown LVS. These results suggest that previous systems may not be directly relevant to the conditions occurring during *in vivo* infection and prompted the notion that Ft might change in response to the immediate environment.

We have pursued this notion and shown that Ft grown in BHI media takes on a host-adapted phenotype and mimics MΦ-grown Ft which is in contrast to Ft cultured in the more commonly used MHB media. Ft differentially expresses virulence factors and capsular material in these different growth conditions, and BHI-grown Ft were found not to stimulate pro-inflammatory cytokine production by MΦs which correlates with the lack of inflammatory response seen clinically at early time points [31,32]. The presence of the capsular material on BHI- and MΦ-grown Ft was found to reduce access of several lipoprotein TLR2 agonists leading to a corresponding decrease in TLR2-dependent, T_H_1 pro-inflammatory cytokine production [32]. Recently, the Bosio group reported that Ft capsule has an additional anti-inflammatory property [33]. Specifically, purified capsule was able to dampen MΦ cytokine resonses to P3C (a synthetic TLR2 agonist) and to an inflammatory, capsule mutant of S4 [33].

Singh et al. [6] used MHB-, BHI-, and MΦ-grown LVS and S4 to delve more deeply and found that the pro-inflammatory nature of MHB-grown wildtype Ft was also attributable to compromised structural integrity of the bacterium [6–8]. Nearly 20% of early-log phase, MHB-grown Ft was found to have compromised membranes that liberated DNase-sensitive material that was recognized in an AIM2-dependent manner to provoke IL-1β secretion by MΦs [6]. Separately, in a confirmation of the genetic results of Peng [29], Singh et al. [6] found that several mutants of Ft that had been postulated to be lacking immunosuppressive genes [34,35] also displayed compromised structural integrity even when grown in BHI. The authors suggested that i) growth of wild type Ft in MHB induced morphological and structural aberrations similar in immunological magnitude to those observed for mutant Ft by the Monack group and ii) that the use of BHI- or MΦ-grown Ft for *in vitro* cell-based assays could bring the results more in line with those observed *in vivo*.

In addition to the compromised physiological state of the bacteria, some host components of infection were also absent in early *in vitro* experiments. Pierini [36] showed that the presence of serum during an *in vitro* infection increased the binding and uptake of LVS into MΦs [36]. This binding and uptake was reduced with heat inactivation of the serum as well as through perturbation of class A scavenger receptors on the MΦs. Dai et al. [37] also showed that the presence of complement protein C3 in serum and complement receptor 3 (CR3) on MΦs resulted in an increase of S4 uptake and a decrease of pro-inflammatory cytokine production likely via lower activation of NF-κB pathways [37]. Significantly, signalling through CR3 was shown to inhibit TLR2 signalling to blunt T_H_1 pro-inflammatory responses to Ft [37]. Whether engagement of CR3 or another “anti-inflammatory” receptor also contributes to the production of the anti-inflammatory cytokines IL-10 and TGF-β, or the lipid mediator PGE2 [38], is currently unknown. Both IL-10 and TGF-β are produced i) *in vitro* by DCs responding to healthy Ft and ii) in early-phase (starting on day 1) pulmonary tularemia where these cytokines contribute to a suppressive milieu involving tolerogenic DCs, regulatory T cells, and myeloid-derived suppressor cells that promote bacterial growth and development of late phase disease [8,39].

Very recently, a novel mechanism of cellular infection by Ft, trogocytosis, was reported which could have implications for both *in vitro* modelling and our broader understanding of tularemia pathogenesis [40]. Previously, it had been understood that Ft replicates within host cells but does not provoke cell-to-cell spreading via actin-based motility as shown for *Listeria* and *Shigella*. Consequently, extracellular Ft that were liberated from exhausted host cells were presumed to interact with new host cells as an encapsulated bacterium that would engage the surface of the new cell. In trogocytosis, one cell donates cytoplasmic contents (potentially including Ft) to an adjacent cell during transient membrane-fusion contacts. In this means of transmission, the bacterium bypasses the surface of the recipient cell and directly enters the cytoplasm with no extracellular intermediate. While the cytokine/immunological consequences of Ft trogocytosis have not yet been reported, it is likely to be a stealthy mode of intracellular travel.

Taken together, these above results suggest that *in vitro* assays that utilize host-adapted Ft and include host components, more accurately model *in vivo* infection and may explain the initial lack of T_H_1 immune stimulation.

## Conclusions

Pneumonic tularemia is characterized by a lack of an early T_H_1 inflammatory response and the presence of an early T_H_17 response accompanied by the production of anti-inflammatory cytokines. This is followed in the latter stages of disease by a septic, T_H_1 cytokine storm and acute onset of symptoms that are likely due to severe tissue damage and release of host danger signals. The mechanisms behind this course of disease are currently being investigated and both *in vitro* and *in vivo* model systems have evolved to account for the involvement of host factors and host-adapted bacteria. It has been suggested that Ft is actively suppressing the immune response at early time points of infection; this was supported by Ft inhibition of an exogenous pro-inflammatory response and several mutant strains of Ft indicated possible effector proteins responsible for the inhibition. However, further investigation of the mutant strains revealed that loss of their structural integrity was likely driving the increased inflammatory response as opposed to the mutated proteins no longer actively repressing inflammatory signalling. In addition, Ft inhibition of exogenous pro-inflammatory signals is likely explained, to some extent, by the bacterial capsule and a stealth-like interaction with host serum factors and anti-inflammatory surface receptors. While the lack of pro-inflammatory stimulation is largely attributable to bacterial host-adaptation and the engagement of anti-inflammatory host factors, the action of potential secreted Ft effector proteins cannot be excluded and further investigation of Ft is on-going in an effort to characterize and better understand host responses to this the deadly infectious agent.
